# Cutaneous HPV23 E6 Prevents p53 Phosphorylation through Interaction with HIPK2

**DOI:** 10.1371/journal.pone.0027655

**Published:** 2011-11-16

**Authors:** Dorothea Muschik, Ilona Braspenning-Wesch, Eggert Stockfleth, Frank Rösl, Thomas G. Hofmann, Ingo Nindl

**Affiliations:** 1 Viral Skin Carcinogenesis Group, Division Viral Transformation Mechanisms, German Cancer Research Center (DKFZ), DKFZ–Charité, Heidelberg, Germany; 2 Department of Dermatology, Venereology and Allergy, Skin Cancer Center Charité, Charité, University Hospital of Berlin, Berlin, Germany; 3 Cellular Senescence Group, German Cancer Research Center (DKFZ), DKFZ-ZMBH Alliance, Heidelberg, Germany; Maastricht University Medical Center, The Netherlands

## Abstract

Ultraviolet irradiation (UV) is the major risk factor for the development of skin cancer. Moreover, increasing evidence suggests cutaneotropic human papillomaviruses (HPV) from the beta genus to play a causal role as a co-factor in the development of cutaneous squamous cell carcinoma. Homeodomain-interacting protein kinase 2 (HIPK2) operates as a potential suppressor in skin tumorigenesis and is stabilized by UV-damage. HIPK2 is an important regulator of apoptosis, which forms a complex with the tumor suppressor p53, mediating p53 phosphorylation at Ser 46 and thus promoting pro-apoptotic gene expression. In our study, we demonstrate that cutaneous HPV23 E6 protein directly targets HIPK2 function. Accordingly, HPV23 E6 interacts with HIPK2 both *in vitro* and *in vivo*. Furthermore, upon massive UVB-damage HPV23 E6 co-localizes with endogenous HIPK2 at nuclear bodies. Functionally, we demonstrate that HPV23 E6 inhibits HIPK2-mediated p53 Ser 46 phosphorylation through enforcing dissociation of the HIPK2/p53 complex. In addition, HPV23 E6 co-accumulates with endogenous HIPK2 upon UV damage suggesting a mechanism by which HPV23 E6 keeps HIPK2 in check after UV damage. Thus, cutaneous HPV23 E6 prevents HIPK2-mediated p53 Ser 46 phosphorylation, which may favour survival of UV-damaged keratinocytes and skin carcinogenesis by apoptosis evasion.

## Introduction

Human papillomaviruses (HPV) infect keratinocytes in the mucosa and the skin. Presently, 151 HPV types are completely characterized (http://pave.niaid.nih.gov) and classified into mucosal/genital (alphaPV) and cutaneous (betaPV, gammaPV, muPV, and nuPV) HPV types, based on sequence analyses and clinical manifestation [1]–[3]. HPV DNA is present in various epithelial tumors and human high-risk genital HPV types (mainly HPV16 and HPV18) are the main risk factors as well as etiologically involved in the development of cervical cancer [4]–[6]. The oncogenes E6 and E7 of genital high-risk HPV are consistently expressed in cervical cancer and the inactivation of the tumor suppressor proteins p53 by E6 and the retinoblastoma protein (pRb) by E7 have been recognized as important pathogenic mechanisms of tumor formation [7]. The E6 oncogenes of genital high-risk HPV bind E6AP promoting the ubiquitin-mediated degradation of p53, an important key regulator of the cell cycle and apoptosis in DNA damaged cells [8], [9].

The first evidence for the involvement of specific HPV types (betaPV) in cutaneous squamous cell carcinoma (SCC) was reported in *Epidermodysplasia verruciformis* (EV) patients. EV is a rare hereditary disease that pre-disposes individuals to cutaneous HPV infections (mainly HPV5 and HPV8), being present in 90% of SCC [10], [11]. Ultraviolet (UV) radiation is the major risk factor for skin cancer. Carcinogenesis is a multi-step process and a co-carcinogenic role of cutaneous human betaPV and SCC was reported for long-term immunosuppressed patients (e.g. organ transplant recipients) and immunocompetent individuals in functional and epidemiological studies [12]–[14]. A higher viral load of betaPV in actinic keratosis (AK) compared to SCC suggests a role of cutaneous HPV in the early stages of skin cancer [15]. HPV23 (beta2PV) is the most prevalent type detected in the skin of immunosuppressed and immunocompetent individuals [16]–[18]. In functional studies, the most examined cutaneous HPV types belong to beta1PV (e.g., HPV5, HPV8, and HPV20) followed by beta2PV (e.g., HPV20, and HPV38) [14].

The E6 and E7 proteins of HPV38 display a transforming activity by increasing the life span of human primary keratinocytes (HPK) and by binding pRb with a similar efficiency as HPV16 E7 [19]. The E6 oncogenes of cutaneous HPV types do not bind and degrade p53 indicating that the molecular mechanisms of apoptosis evasion differ between cutaneous and genital HPV types. It has been demonstrated that E6 from some cutaneous HPV types degrade activated pro-apoptotic Bak protein in UV damaged cells thus protecting keratinocytes from apoptosis [20]–[22]. However, mechanisms by which other cutaneous HPV types, such as the most prevalent type HPV23, may interfere with the cellular apoptosis response and therefore might contribute to development of SCC are at present unclear.

The serine/threonine homeodomain-interacting protein kinase 2 (HIPK2) is a key regulator of stress-induced apoptosis [23], [24]. HIPK2 is activated and stabilized in UV-induced DNA damaged cells by the ATM/ATR pathway [25]. Upon UV-induced severe DNA damage, HIPK2 binds to p53 and phosphorylates p53 at serine 46 (Ser 46), which stimulates p53 stabilization, CBP-mediated p53 acetylation and transcriptional activation of pro-apoptotic factors such as Bax and p53AIP1 [23], [24], [26], [27]. HIPK2-mediated p53 Ser 46 phosphorylation presumably takes place at promyelocytic leukemia (PML) nuclear bodies. Nuclear domains play an important role in antiviral response and cell fate regulation [28]. Recently, it has been demonstrated that genetic deletion of HIPK2 in mice potentiates skin tumorigenesis induced by the two-stage carcinogenesis protocol [29], showing that HIPK2 acts as a tumor suppressor in the skin.

In the present study, we investigated a potential link between E6 from cutaneous HPV types with the tumor suppressor HIPK2. We show that the E6 protein of the most prevalent cutaneous type HPV23, physically interacts with HIPK2 both *in vitro* and *in vivo*, and that HPV23 E6 co-localizes with HIPK2 in nuclear bodies. Moreover, we demonstrate that binding of E6 to HIPK2 inhibits HIPK2-mediated p53 Ser 46 phosphorylation by enforcing dissociation of the HIPK2/p53 complex. Therefore, our findings suggest a novel mechanism by which cutaneous HPV types prevent the UV-activated cell death response and thus may contribute to skin carcinogenesis.

## Results

### *In vitro* and *in vivo* interaction of cutaneous HPV23 E6 with HIPK2

**Figure 1:**
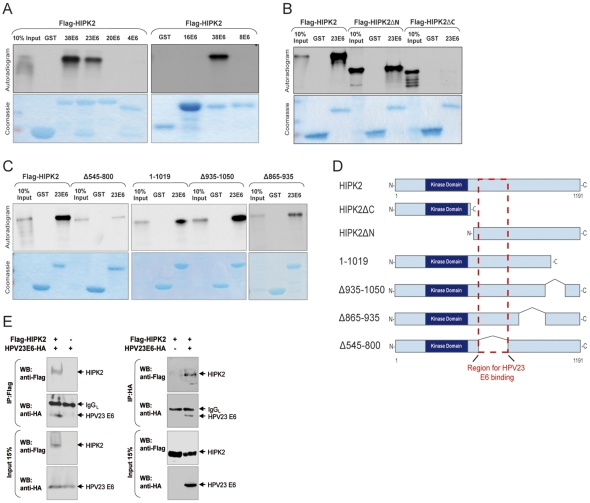
HIPK2 and HPV23 E6 proteins interact *in vitro* and *in vivo*. (**A–C**) GST and various GST-tagged HPV E6 proteins were incubated with *in vitro* translated ^35^S-labelled HIPK2 protein. GST was used as a negative internal control. GST pull-down experiments were analyzed by SDS–PAGE and stained with Coomassie brilliant blue to estimate the protein concentrations (lower panels). One-tenth of the total ^35^S-labelled HIPK2 input was applied to the SDS-PAGE. Gels were dried and exposed to X-ray films and autoradiograms are shown in the upper panels. (**A**) HPV38 E6 and HPV23 E6 proteins (cutaneous beat2PV) interact with HIPK2. HPV16 E6 (alphaPV), HPV4 E6 (gammaPV), HPV8 E6, and HPV20 E6 (cutaneous beta1PV) failed to physically interact with HIPK2. (**B**) HPV23 E6 proteins interact mainly with the C-terminus of HIPK2 (HIPK2ΔN). (**C**) HPV23 E6 proteins showed reduced binding to the C-terminal deletion mutant Δ545–800 of HIPK2. (**D**) Schematic presentation illustrates the full-lenght and deletion mutants of HIPK2, analyzed in GST pull-down experiments. The HIPK2 kinase domain and the predicted HPV23 E6 binding region are indicated. (**E**) HPV23 E6 bind *in vivo* to HIPK2 in H1299 cells, which were transfected with HA-tagged HPV23 E6 and Flag-tagged HIPK2 either alone or in combination and immunoprecipitated (IP) with Flag (M2) or HA (clone 12CA5) antibodies. Immunoprecipitated protein complexes were analyzed by Western blot (upper panels). The light chain of the precipitating antibody (IgG_L_) is indicated. In the lower panels the expressed proteins levels of HIPK2 and HPV23 E6 in H1299 cells are shown.

Cutaneous HPV E6 proteins (beta1PV types) effectively inhibit apoptosis in response to UV damage [21]. Since the kinase HIPK2 is an important tumor suppressor within the skin and regulates UV-damage induced apoptosis by activating p53 [24], [25], we hypothesized that cutaneous HPV E6 proteins may interact with this key apoptotic kinase. To examine an interaction of HIPK2 with E6 proteins of genital and cutaneous HPV types, glutathione S-transferase (GST) pull-down experiments were performed. HIPK2 was labelled with ^35^S-Methionin by *in vitro* transcription/translation and tested for its binding with various purified GST–HPV E6 fusion proteins of different types (Fig. 1). Only E6 of beta2PV types (HPV23, and HPV38) physically bind HIPK2 *in vitro,* whereas no binding was observed with genital alphaPV (HPV16), beta1PV (HPV8, and HPV20) and gammaPV (HPV4) types (Fig. 1A). These results indicate a specific interaction between HIPK2 and cutaneous HPV E6 proteins of beta2PV types.

Various HIPK2 deletion mutants were used to map the region required for the binding between HIPK2 and E6 of HPV23. The C-terminus of HIPK2 (HIPK2ΔN) was able to bind E6 of HPV23. In contrast, virtually no binding was observed with the N-terminus (HIPK2ΔC) (Fig. 1B). Furthermore, no major difference in the binding efficiency was detected with the C-terminal HIPK2 deletion mutants HIPK2 1–1019, HIPK2 Δ925–1050, and HIPK2 Δ865–935. However, reduced interaction between HIPK2 and E6 was evident when amino acids 545–800 were deleted from HIPK2 suggesting a role of this region for E6 binding (Fig. 1C). Figure 1D show a schematic illustration of HIPK2 and all HIPK2 deletion mutants, which we have analyzed for *in vitro* bindings with HPV E6 proteins.

To further examine *in vivo* interaction of HPV23 E6 and HIPK2, we co-transfected mammalian H1299 cells with HA-tagged E6 of HPV23 and Flag-HIPK2 (Fig. 1E). Co-immunoprecipitation analysis revealed that HIPK2 interacts with HPV23 E6 protein *in vivo*. Similar results were obtained with the kinase-deficient mutant HIPK2^K221A^ (Fig. S1). Altogether, the results identify an interaction between HPV23 E6 and the apoptotic regulator HIPK2.

### HIPK2 co-localize with HPV23 E6 proteins in nuclear bodies

**Figure 2:**
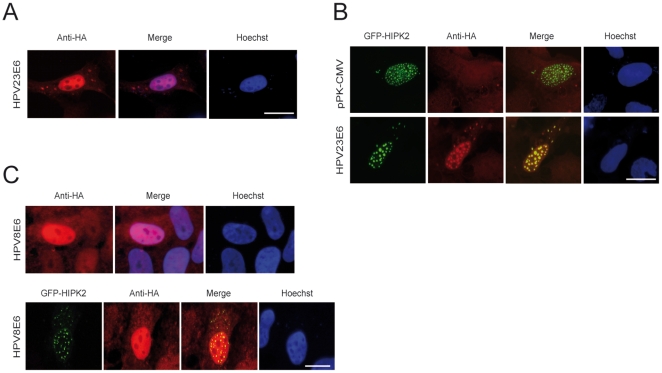
HIPK2 co-localize with HPV23 E6 protein in nuclear bodies. U2OS cells were transfected with HA-tagged HPV E6 either alone or in combination with GFP-HIPK2 and examined by immunofluorescence microscopy. Nuclear DNA (blue) was stained with Hoechst dye. Scale bars represent 10 µm. (**A**) Note diffuse expression patterns of HPV23 E6 proteins and (**B**) nuclear re-localization of E6 and co-localization (yellow) of GFP-HIPK2 (green) and E6 proteins (red) in nuclear bodies. (**C**) No re-localization of HPV8 E6 after overexpression of HIPK2 was observed and HIPK2 (green) do not co-localize with HPV8 E6 protein (red) demonstrated by the diffuse pattern of E6 expression.

Previous studies reported a diffuse HPV E6 protein pattern of mucosal (alphaPV) and cutaneotropic (betaPV) HPV types in the nucleus [30]–[32]. Next we examined the subcellular localization of HPV23 E6 and HPV8 E6 in the presence and absence of overexpressed HIPK2. U2OS cells were transfected with plasmids containing HA-tagged E6 and/or GFP-tagged HIPK2, fixed and stained with anti-HA antibodies. Immunofluorescence microscopy showed that the E6 proteins of HPV23 and HPV8 had a diffuse expression pattern in the nucleus (Fig. 2A and 2C). In the presence of GFP-tagged HIPK2, HPV23 E6 co-localized with HIPK2 and was targeted to nuclear bodies (Fig. 2B), whereas HPV8 E6 did not co-localize with HIPK2 (Fig. 2C). Taken together, these data indicate that HIPK2 mainly targets HPV23 E6 to nuclear bodies.

### Lethal UVB radiation induces co-localization of HPV23 E6 with HIPK2 in nuclear bodies

**Figure 3:**
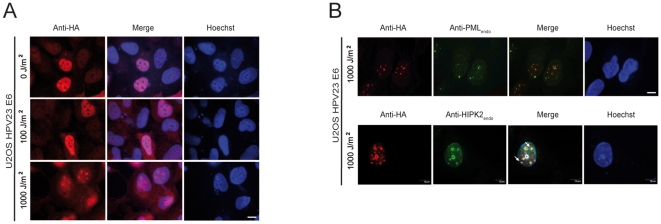
Lethal UVB radiation induces re-localization of HPV23 E6 proteins and co-localization with HIPK2. (**A–B**) Stably expressing HA-tagged HPV23 E6 U2OS cells were treated with sub-lethal (100 J/m^2^) or lethal (1,000 J/m^2^) UVB doses. Nuclear DNA (blue) was stained with Hoechst dye. Scale bars represent 10 µm. (**A**) Immunofluoresence staining and microscopy show a diffuse expression pattern of HPV23 E6 proteins in untreated or treated (100 J/m^2^ UVB) cells (red). Re-localization in nuclear bodies of HPV23 E6 proteins (red) was observed after lethal UVB doses (1,000 J/m^2^). (**B**) These nuclear bodies did mainly not co-localize with endogenous promyelocytic leukaemia nuclear bodies (PML bodies) (green, upper panels) but co-localize with endogenous activated HIPK2 protein (green) in U2OS cells after lethal UVB radiation (yellow, lower panels, white arrows).

Cutaneous HPV seems to be a co-factor in the development of cutaneous SCC in association with UVB irradiation [13], [14]. HIPK2 is activated and stabilized in DNA-damaged cells, phosphorylate p53 at Ser 46, which leads to the induction of apoptosis [24]. To examine the localization of HPV23 E6 after UVB-induced activation of endogenous HIPK2, we irradiated stably HPV23 E6 expressing U2OS cells using both sub-lethal (100 J/m^2^) and lethal (1,000 J/m^2^) UVB doses. HPV23 E6 remained localized diffusely in the cell nucleus both in undamaged and sub-lethally UV-damaged cells (Fig. 3A) similar to the observed pattern in Figure 2A. In contrast, after lethal UVB irradiation, HPV23 E6 re-localized into nuclear bodies (Fig. 3A). Moreover, HPV23 E6 co-localized with endogenous HIPK2 protein in nuclear bodies but the E6 positive nuclear bodies did not substantially overlap with PML bodies (Fig. 3B). Taken together, our results indicate that HPV23 E6 co-localizes with HIPK2 upon lethal UV irradiation in nuclear bodies, which appear to be distinct to PML bodies.

### HPV23 E6 inhibits HIPK2-mediated phosphorylation of p53 at Ser 46 by enforced dissociation of the HIPK2/p53 complex

**Figure 4:**
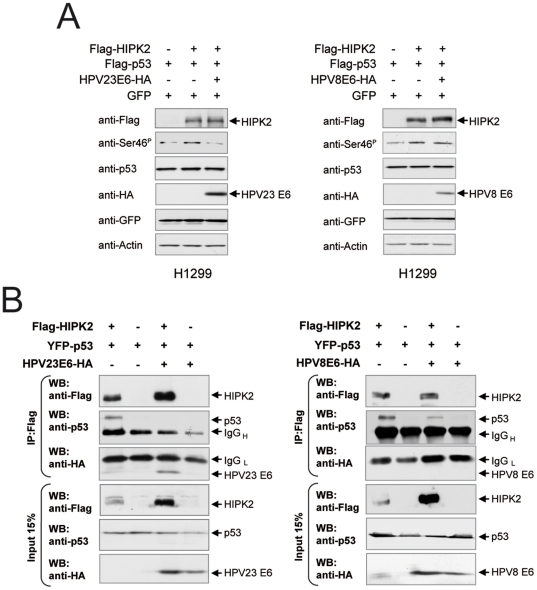
HPV23 E6 proteins dissociate the HIPK2/p53 complex and inhibit HIPK2 phosphorylation of p53 at Ser 46. Western blot analyses of transfected p53-negative H1299 cells with HPV23 E6-HA and/or Flag-HIPK2 and/or Flag-p53 (A and B) or YFP-p53 (B). (**A**) HPV23 E6 expression reduces HIPK2 phosphorylation of p53 at Ser 46 shown by Western blot. HPV8 E6 proteins, which do not bind HIPK2, have no effect on p53 phosphorylation at Ser 46. (**B**) HPV23 E6 proteins dissociated the HIPK2/p53 complex formation. Immunoprecipitation (IP) with Flag antibody (M2) of transfected H1299 cells (see above) to examine *in vivo* binding of HIPK2 with p53 in the presence or absence of HPV23 E6. To examine protein expression in H1299 (lower panels), 15% of cell lysates were analyzed by Western blot. The light and heavy chains of the precipitating antibody (IgG_L_ and IgG_H_) are indicated.

The protein kinase HIPK2 forms a complex with the tumor suppressor p53 upon UV-induced DNA damage, mediating p53 phosphorylation at Ser 46, which leads to the transcriptional activation of pro-apoptotic factors [23], [24]. To asses the functional consequences of the interaction between HPV23 E6 and HIPK2, we analyzed the impact of HPV23 E6 on p53 Ser 46 phosphorylation. We found that HIPK2-induced phosphorylation of p53 at Ser 46 was reduced by HPV23 E6 proteins, but not by the non-binding E6 protein of HPV8 (Fig. 4A).

To obtain insight into the mechanisms underlying the inhibition of HIPK2-mediated p53 Ser 46 phosphorylation by HPV23 E6 we analyzed whether HPV23 E6 has an influence on HIPK2/p53 complex formation. Co-immunoprecipitation assays demonstrated that HPV23 E6 protein enforced the dissociation of the HIPK2/p53 complex (Fig. 4B). In contrast, HPV8 E6, which does not bind to HIPK2, showed only marginal effects on HIPK2/p53 complex formation. Thus, we conclude that cutaneous HPV23 E6 binds HIPK2 and inhibits the HIPK2/p53 complex, thereby preventing p53 Ser 46 phosphorylation.

### HPV23 E6 co-accumulates with HIPK2 upon DNA damage

**Figure 5:**
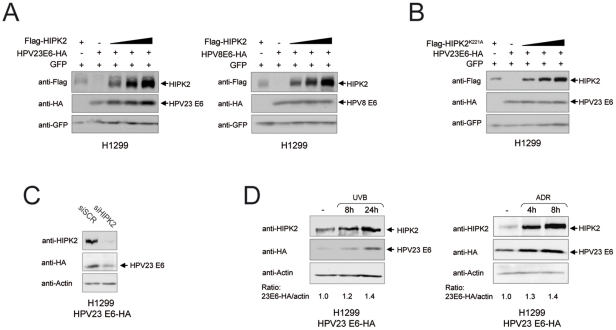
Accumulation of HPV23 E6 protein is caused by HIPK2. (**A–B**) Western blot analyses of H1299 cells, which were co-transfected with HA-tagged E6 of HPV23 (beta2PV) or HPV8 (beta1PV) and/or Flag-tagged HIPK2 and/or the kinase-deficient mutant HIPK2^K221A^. To monitor equal transfection efficiency GFP was used as internal control. (**A**) Increasing HIPK2 levels causes accumulation of exclusively HPV23 E6 proteins, and no accumulation of HPV8 E6 proteins. (**B**) The kinase-deficient mutant HIPK2^K221A^ does not cause accumulation of HPV23 E6 proteins indicating that the kinase activity is essential for E6 protein accumulation. (**C**) Inhibition of HIPK2 expression in stably HPV23 E6 expressing H1299 cells by specific RNAi siHIPK2 (negative control siScr). Reduction of HIPK2 protein caused a decrease of E6 protein levels shown by Western blot analysis. (**D**) Stably HPV23 E6 expressing H1299 cells were treated with lethal UVB doses (1,000 J/m^2^) or adriamycin (0.5 µg/ml; ADR) and examined by Western blot. The amount of E6 proteins was quantified using the Odysse software. Activated endogenous HIPK2 upon DNA damage (8 h and 24 h after UVB-radiation, left panel; or 4 h and 8 h after ADR treatment, right panel) caused accumulation of HPV23 E6 proteins. The quantified amounts of HPV23 E6 proteins are shown as the ratio of HPV23 E6/actin.

To examine a putative protein accumulation or degradation of E6 by HIPK2, we performed co-transfection experiments. We co-transfected H1299 cells with E6 of HPV23 and HPV8 and increasing amounts of HIPK2 including the internal control GFP. We found that HIPK2 specifically triggered accumulation of HPV23 E6, but not HPV8 E6, in a dose-dependent fashion (Fig. 5A). Furthermore, we analyzed the effects of the kinase-deficient mutant HIPK2^K221A^ on HPV23 E6. No increase in E6 protein amounts was detected indicating that the kinase activity is essential for accumulation of HPV23 E6 (Fig. 5B). To examine the effect of endogenous HIPK2 on E6 accumulation, we knocked-down HIPK2 using specific RNAi (siHIPK2) in stably expressing HPV23 E6 cells (Fig. 5C). Strikingly, reduction of HIPK2 leads to a decrease of HPV23 E6 protein showing that E6 accumulation is regulated by endogenous HIPK2. We further verified our observation by inducing stabilization of endogenous HIPK2 in stably expressing HPV23 E6 cells upon DNA damage by UVB-radiation or chemotherapeutical treatment with adriamycin (ADR). Remarkably, HPV23 E6 co-accumulated with endogenous HIPK2 in response to UV- and chemotherapeutic drug-induced DNA damage (Fig. 5D). Since the kinase activity of HIPK2 is essential for HPV23 E6 accumulation after DNA damage, we analyzed whether HIPK2 phosphorylates HPV23 E6. No phosphorylation of HPV23 E6 was observed using in vitro assays suggesting that HPV23 E6 accumulation is not mediated by direct phosphorylation by HIPK2 (Fig. S2). Taken together, these results indicate that HPV23 E6 accumulates along with HIPK2 after UV-damage suggesting that HPV23 E6 is co-accumulated by a HIPK2-mediated feed-forward loop in order to facilitate an efficient negative control of the apoptotic function of HIPK2.

## Discussion

The main risk factor of cutaneous SCC is UV-radiation, which induces DNA damage. HPV seems to be an etiological factor in the early onset of this disease. However, the mechanisms of this viral or non-viral induced skin cancer are mainly unclear. In our present study, we identified that the key kinase HIPK2 is a novel HPV23 E6 interaction partner upon DNA damage. The presence of E6 inhibits the HIPK2/p53 complex and, additionally, posphorylation of p53 at Ser 46, which is a central event in the induction of apoptosis. Our data performed in heterologous cell lines provide a good basis to examine the functional interaction of HPV23 E6 with HIPK2 in cutaneous keratinocytes.

The E6 protein of cutaneous HPV5 (beta1PV) inhibits apoptosis upon UV-induced DNA-damage by binding and degrading Bak protein [20], [21]. Moreover, E6 of the cutaneous HPV types 5, 8, 20, 22, 38, 76, 92, and 96 (betaPV types) degrade Bak or prevent Bak protein accumulation after UV-induced DNA damage resulting in the protection of apoptosis [22] demonstrating that E6 have anti-apoptotic properties. The kinase HIPK2 is stabilized upon UV-induced DNA damage, forms a complex with p53 in nuclear bodies and phosphorylates p53 at Ser 46 and thus, is a key regulator of the apoptotic program [25]. Mice lacking HIPK2 expression fastly developed skin cancer using the two step skin carcinogenesis protocol indicating that HIPK2 is a tumor suppressor gene and involved in cutaneous SCC [29]. In our study, we found that E6 proteins of cutaneous HPV types bind HIPK2 both *in vitro* and *in vivo*. Interestingly, only E6 of beta2PV types (HPV23 and HPV38) but not beta1PV types (HPV8 and HPV20), cutaneous gammaPV (HPV4) or genital HPV16 were able to bind HIPK2. Thus, the molecular mechanism of at least one central pathway differs in the beta genus of cutaneous HPV types. HPV38 but not HPV20 showed transforming properties in human primary keratinocytes [19] indicating that beta2PV types probably have a higher oncogenic risk to develop skin cancer compared to beta1PV types.

It has been previously demonstrated that E6 proteins of the cutaneous HPV types 12, 14, 24 and 49 of the beta genus localized largely to the nucleus of U2OS cells, and no significant co-localization with nuclear PML bodies was observed [32]. We examined the localization of E6 proteins of HPV23 and HPV8 in the presence and absence of HIPK2 in U2OS cells and we also observed a diffuse E6 protein expression pattern of both types in the nucleus. In the presence of transiently expressed HIPK2, we found a co-localization of HPV23 E6 with HIPK2 in nuclear bodies demonstrating that only HPV23 E6 forms a complex with HIPK2 but not HPV8 E6.

HPV23 E6 proteins were not a direct target of HIPK2 because no phosphorylation of HPV23 E6 was observed using *in vitro* assays (see Fig. S2). Since UV-radiation is the first event in skin carcinogenesis, we activated endogenous HIPK2 with different UV doses inducing DNA-damage and observed complex formation of HIPK2 with HPV23 E6 but not HPV8 E6.

It was shown that after DNA damage a fraction of HIPK2 is recruited to PML bodies where HIPK2 associated with p53 and phosphorylate p53 at Ser 46 resulting in the induction of apoptosis [23], [24], [28]. We observed no co-localization of the HIPK2/HPV23 E6 nuclear complex with PML bodies indicating that probably the apoptotic function of HIPK2 in the presence of E6 is inhibited. Indeed HPV23 E6, but not HPV8 E6 prevents phosphorylation of p53 at Ser 46 by HIPK2 in H1299 cells.

**Figure 6:**
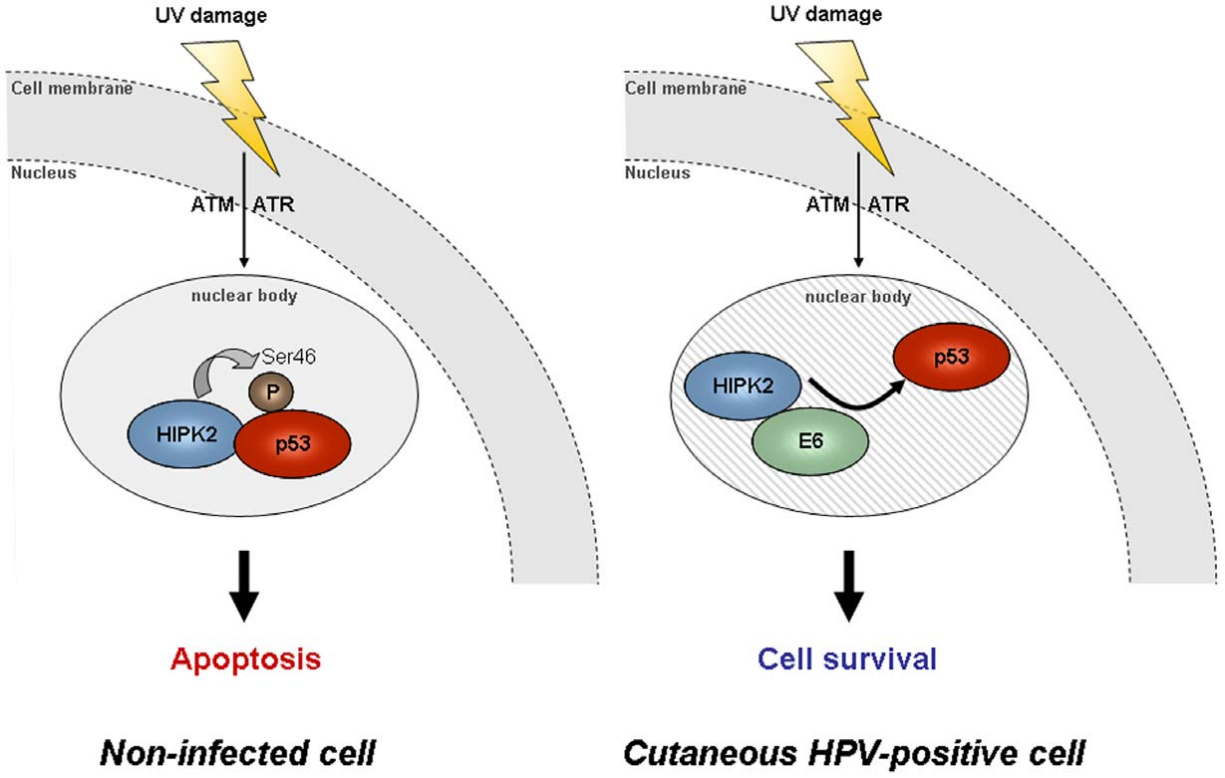
Proposed model for HPV23 E6 inhibition of the HIPK2/p53 complex upon UV-induced DNA-damage. In response to DNA damage, checkpoint kinases ATM and/or ATR mediate HIPK2 stabilization and activation**.** In response to lethal DNA damage, HIPK2 activates apoptosis by phosphorylation of p53 at Ser 46 in nuclear bodies. In HPV infected cells, HPV23 E6 binds HIPK2 and dissociates the HIPK2/p53 complex. E6 binding to HIPK2 inhibits the phosphorylation of p53 at Ser 46.

Moreover, complex formation of HIPK2 with p53, which is critical for p53 Ser 46 phosphorylation [33], was inhibited in the presence of HPV23 E6 (Fig. 6). Since the p53 Ser 46 phosphorylation mark is essential for UV-induced apoptosis induction [24], our results suggest a possible mechanism of anti-apoptotic features of the HPV23 E6 protein.

Direct or indirect degradation of HIPK2 by HPV23 E6 would be a putative mechanism to inhibit apoptosis upon UV-induced DNA-damage. Our results do not indicate that HIPK2 is degraded by HPV23 E6. Interestingly, in contrast, our findings indicate that HPV23 E6 uses HIPK2 activity to trigger its own accumulation. These results suggest a feed-forward regulatory mechanism between HIPK2 and HPV23 E6, which facilitates co-accumulation of HPV23 E6 in parallel of HIPK2 upon UV damage. Such a mechanism might provide an efficient means to counteract the apoptotic activity of HIPK2 upon its UV-induced stabilization.

In conclusion, HPV23 E6 interacts with HIPK2 upon UV-induced DNA-damage inhibiting HIPK2/p53 complex formation, the key event in the induction of UV-induced apoptosis. Thus, cutaneous HPV infected keratinocytes may overcome UV-induced apoptosis and be causal in the early onset of skin carcinogenesis.

## Materials and Methods

### Cell lines

Cells were incubated in a humidified 5% CO_2_ atmosphere at 37°C. Human osteosarcoma cell line U2OS (p53 wild type) [34] and non-small lung carcinoma cell line H1299 (p53 negative) (obtained from ATCC) were cultured in Dulbecco's modified Eagle's medium (DMEM) with 4.5 g of glucose per liter and L-glutamine, supplemented with 10% fetal bovine serum. The cells were transiently or stably transfected, using Lipofectamine 2000 (Invitrogen) according to the manufacturer's instructions. For the stable transfection, cells were additionally selected with 850 µg/ml G418 containing medium for more than two weeks.

### UVB irradiation and adriamycin treatment

Cells were allowed to reach a 50% confluence. The medium was removed and the cells were washed once with PBS and irradiated with experimental determined UVB radiation doses. We have used either 100 J/m^2^ (sublethal dose) or 1,000 J/m^2^ (lethal dose) of UVB (Waldmann UV181 BL) with an output range of 280 to 320 nm. The amount of UVB energy was measured with an UVB detector. After UVB treatment the cells were incubated within a fresh medium and harvested at the time points indicated. For adriamycin (ADR) treatment, cells were allowed to reach a minimum of 50% confluence. The medium was supplemented with ADR (0.5 µg/ml) and the cells were harvested at the indicated times points.

### Expression vectors

N-terminal Flag-tagged HIPK2 plasmids expressing full-length wild-type HIPK2 and HIPK2 deletion mutants have been previously described [24], [35]. The pGEX plasmid was used to generate N-terminal GST-tagged E6 fusion proteins from different HPV types. The plasmids pGEX-HPV16 E6 (alphaPV), pGEX-HPV8 E6 (beta1PV) and pGEX-HPV38 E6 (beta2PV) were a gift from M. Pawlita (DKFZ, Heidelberg, Germany). The E6 ORF of HPV4 (gammaPV), HPV20 (beta1PV), and HPV23 (beta2PV) were amplified by PCR with BamHI/SalI recognition sites and cloned into the vector pGEX-4T3 or pGEX-4T1 (GE Healthcare), designated pGEX-HPV4E6, pGEX-HPV20E6, and pGEX-HPV23E6. The following primers were used: HPV4E6_sense 5′-ATACGGATCCATGGCAGATGGC-3′ and HPV4E6_antisense 5′-GTCAGTCGACTTGTTTCCTAATACAA-3′, HPV20E6_sense 5′-ATACGGATCCATGGCTACACCT-3′ and HPV20E6_antisense 5′- GTCAGTCGACTTGAAAATGCTT-3′, HPV23E6_sense 5′-ATACGGATCCATGCAGACTGTG-3′ and HPV23E6_antisense 5′- GTCAGTCGACTTCTATTTCCTTACAA-3′. C-terminal Ha-tagged E6 proteins of HPV23 and HPV38 (both beta2PV), and HPV8 (beta1PV) were generated by PCR with EcoRI/BamHI recognition sites and cloned into the plasmid pPK-CMV-E3 (Promokine). The E6 ORF were amplified with the primers HPV23E6_sense 5′- TGACGAATTCACCATGCAGACTGTGCATTATTTAAGTAG-3′ and HPV23E6_antisense 5′- TGACGGATCCTTCTATTTCCTTACAATGCCTGCACCT-3′, HPV38E6_sense 5′- TGACGAATTCACCATGGAACTACCAAAACCTCAAACTG-3′ and HPV38E6_antisense 5′-TGACGGATCC TTCTATTGCTTTGCAATGCCTGCACCT-3′, HPV8E6_sense 5′-TGACGAATTCACCATGGACGGGCAGGACAAGGCTTCA-3′ and HPV8E6_antisense 5′-TGACGGATCCCCAATCATGATACAAATGCTTACAAAG-3′. All plasmids were sequenced and contained the prototype E6 ORF (accession numbers: HPV16, NC_001526; HPV4, NC_001457; HPV8, M12737; HPV20, U31778; HPV23, U31781; and HPV38, U31787).

### Transfection of siRNA

For specific knockdown experiments, siRNA against HIPK2 (siHIPK2: 5′-AACACCTACGAGGTCTTAGAG-3′) [36] and control siRNA (siScramble: 5′-AACAGTCGCGTTTGCGACTGG-3′) [37] were transfected using Dharmafect2 transfection reagent (Dharmacon) according to the manufacturer instructions. One day prior of transfection 50,000 H1299 cells were seeded in each well of 6-well plate and grown to a density of approximately 70% confluence. The transfection mix of 7.5 µl siRNA (40 µM), 10 µl Dharmafect2 and 1.6 ml of DMEM was dropped onto the seeded cells. The growth media was renewed 24 h after transfection followed by a repeated transfection after 48 h. The transfected cells were harvested after 96 h and analyzed by Western blot.

### Antibodies

The following antibodies were used: p53 (DO-1) and PML (PG-M3) from Santa Cruz Biotechnologies, Flag (M2) and GFP (FL) from Sigma, actin (C4) from MP Biomedicals; HA (clone 12CA5 and clone 3F10) from Roche; p53 phospho-Ser46 from Cell Signaling. The affinity-purified HIPK2 antibodies have been previously described [24].

### Immunofluorescence

At 48 h after transfection, U2OS cells were fixed in 3.7% paraformaldehyde in PBS for 20 min at room temperature (RT), washed once with 0.1 M glycine, permeabilized with 0.5% triton X-100/PBS and then incubated with 1% BSA/PBS for 1 h. The primary antibody was diluted in 1% BSA/PBS and incubated for 90 min at RT. HA-tagged proteins were detected with the monoclonal mouse antibody (1∶150; clone 12CA5), endogenous PML proteins with the monoclonal mouse antidody (1∶150; PG-3) and endogenous HIPK2 with the polyclonal antibody (1∶100; C3). After three washing steps in PBS, the cells were incubated with a 1∶450 diluted solution of fluorescein-conjugated goat antibodies (Alexa Fluor® 594, Molecular Probes) for 45 min at RT and additionally incubated with Hoechst staining solution (1∶10,000) for 20 min at RT. Afterwards, the cells were washed several times in PBS and water, then mounted on glass slides. Confocal images were prepared using a FluoView2000 (Olympus) microscope. Images were processed with the AdopePhotoshop software.

### GST pull-down assays

Full-length HIPK2 and different HIPK2 deletion mutants were *in vitro* transcribed and translated using the TNT coupled reticulocyte lysate system (Promega) and radioactive labeled with ^35S^Methionin. The GST protein and GST-E6 fusion proteins of different HPV types were isolated from the bacterium *Escherichia co*li after sonification and centrifugation of the lysate. Protein purification and GST pull-down assays were performed as previously described [24].

### *In vitro* phosphorylation assay

The *in vitro* phosphorylation assay was performed as earlier described [24]. Briefly, for the *in vitro* phosphorylation assay, His-tagged Flag-HIPK2 protein was expressed in *Escherichia co*li and purified using beads. Kinase reactions were performed with 200 ng HIPK2, 1 µg HPV23 GST-E6 fusion proteins or myelin basic protein (MBP) using 30 µl 2-fold kinase buffer containing 40 µM cold ATP and 5 µCi [gamma-^32^P] ATP. After incubation for 30 min at 30°C, the reaction was stopped by adding 5×SDS loading buffer. After separation by SDS–PAGE, gel was fixed with Coomassie, then dried and exposed to X-ray films.

### Co-immunoprecipitation and Western blotting

Entire cell extracts were compounded with denaturing lysis buffer (20 mM Tris, 1% NP40, 150 mM NaCl, 5 mM EDTA, 10% glycerol, 1% SDS) or non-denaturing buffer (20 mM HEPES, 150 mM NaCl, 5 mM EDTA, 10% glycerol, 0.5% Triton-X-100) to perform co-immunoprecipitation and Western blot. Co-immunoprecipitation was performed after cell lysis in non-denaturing buffer. After centrifugation, and purification with Protein-A/G–Sepharose beads, 5 µg Flag (M2) or HA (clone 12CA5), antibody was added to the supernatant and incubated for 3 h at 4°C on a rotating wheel. After incubation, Protein-A/G–Sepharose beads were added for 1 h at 4°C on a rotating wheel. The beads were washed three times in an ice cold non-denaturing buffer. Proteins bound to the antibodies were eluted by boiling for 5 min in 1x SDS sample buffer. Western blot analysis was performed with protein extracts separated by SDS-PAGE and transferred to hyperbond-P transfer membranes (Millipore). Membranes were blocked with 5% non-fat milk or BSA and incubated with different primary antibodies. Western blots were developed using peroxidase-conjugated secondary antibodies and enhanced chemoluminescence substrates (Amersham).

## Supporting Information

Figure S1 **Binding of HPV23 E6 with the kinase-deficient mutant HIPK2^K221A^.** (**A**) GST (negative control) and GST-tagged E6 proteins of HPV23 and HPV38 were incubated with *in vitro* transcribed/translated ^35^S-labelled HIPK2 or the kinase-deficient mutant HIPK2^K221A^. GST pull-down experiments were analyzed by SDS–PAGE, which were stained with Coomassie brilliant blue. The gel was dried and exposed to X-ray film and an representative autoradiogram is shown in the upper panel. E6 of both beta2PV types (HPV23 and HPV38) were able to bind HIPK2 and HIPK2^K221A^. (**B**) *In vivo* binding of HPV23 E6 and HIPK2^K221A^. H1299 cells were transfected with HA-tagged HPV23 E6 and Flag-tagged HIPK2^K221A^ either alone or in combination and immunoprecipitated (IP) with Flag (M2) or HA (clone 12CA5) antibodies. Protein-complexes were analyzed by Western blot (upper panels). The input control (10% cell lysates) was analyzed to monitor expressed protein by Western blot (lower panels). HIPK2^K221A^ was able to co-immunoprecipitate HPV23 E6 (left site) and vice versa (right site).
(TIF)

Figure S2 **HIPK2 does not phosphorylate HPV23 E6.** Autoradiogram showing an *in vitro* HIPK2 kinase assay using GST-tagged HPV23 E6 protein. The target protein MBP of HIPK2 was used as a positive control and GST as a negative control. Phosphorylated MBP is shown in short and long exposition whereas GST and HPV23 E6 was not phosphorylated by HIPK2 under these conditions. The protein loading was monitored by Coomassie blue staining (lower panel).
(TIF)
