# Supplementary figures and images for: Cutaneous HPV23 E6 Prevents p53 Phosphorylation through Interaction with HIPK2
Source: PLoS One. 2011 Nov 16;6(11):e27655. doi: 10.1371/journal.pone.0027655 (PMC3218003; doi:10.1371/journal.pone.0027655)

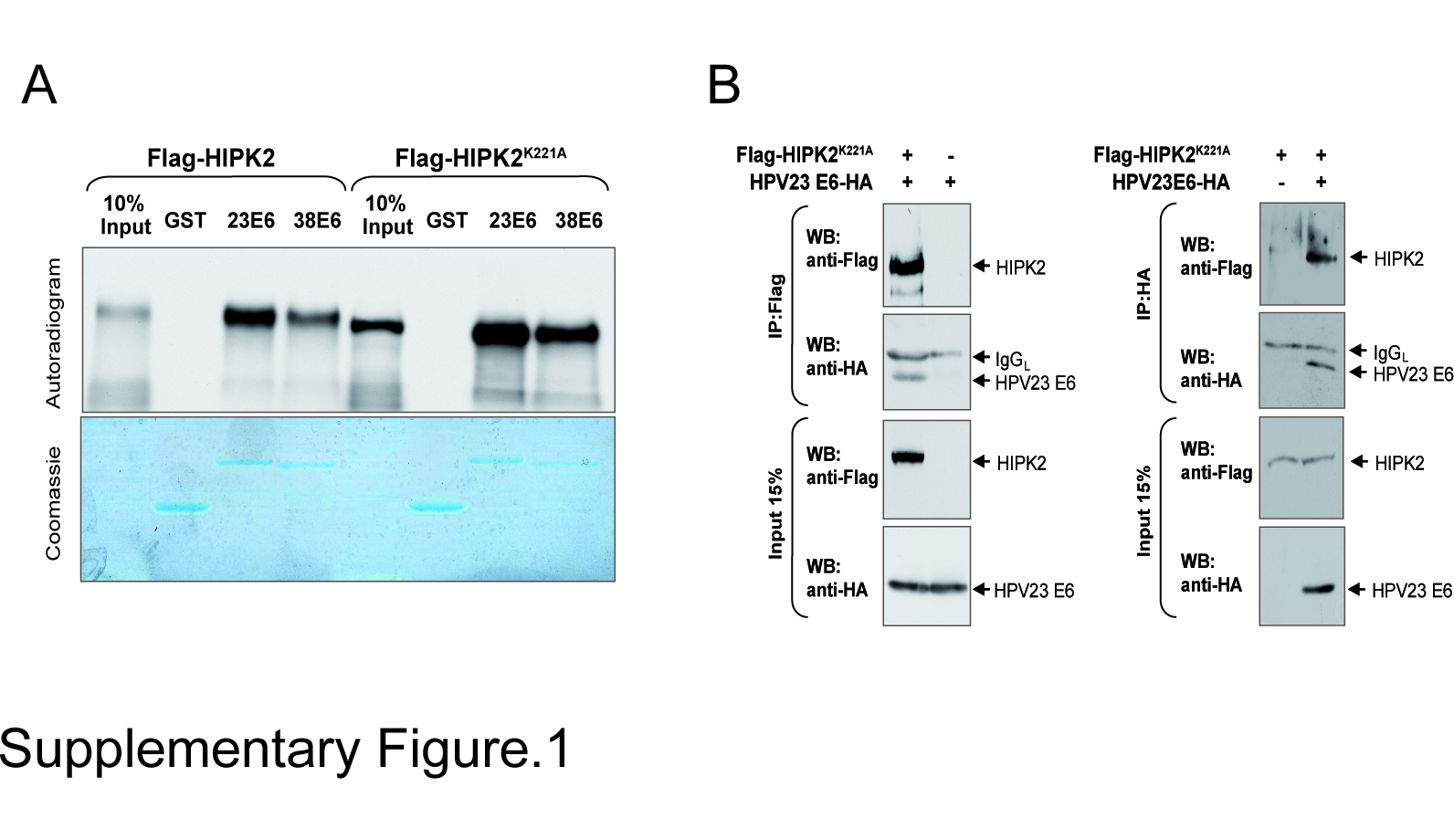

Supplement: Figure S1 — Binding of HPV23 E6 with the kinase-deficient mutant HIPK2K221A. (A) GST (negative control) and GST-tagged E6 proteins of HPV23 and HPV38 were incubated with in vitro transcribed/translated 35S-labelled HIPK2 or the kinase-deficient mutant HIPK2K221A. GST pull-down experiments were analyzed by SDS–PAGE, which were stained with Coomassie brilliant blue. The gel was dried and exposed to X-ray film and an representative autoradiogram is shown in the upper panel. E6 of both beta2PV types (HPV23 and HPV38) were able to bind HIPK2 and HIPK2K221A. (B) In vivo binding of HPV23 E6 and HIPK2K221A. H1299 cells were transfected with HA-tagged HPV23 E6 and Flag-tagged HIPK2K221A either alone or in combination and immunoprecipitated (IP) with Flag (M2) or HA (clone 12CA5) antibodies. Protein-complexes were analyzed by Western blot (upper panels). The input control (10% cell lysates) was analyzed to monitor expressed protein by Western blot (lower panels). HIPK2K221A was able to co-immunoprecipitate HPV23 E6 (left site) and vice versa (right site). (TIF) [file pone.0027655.s001.tif]

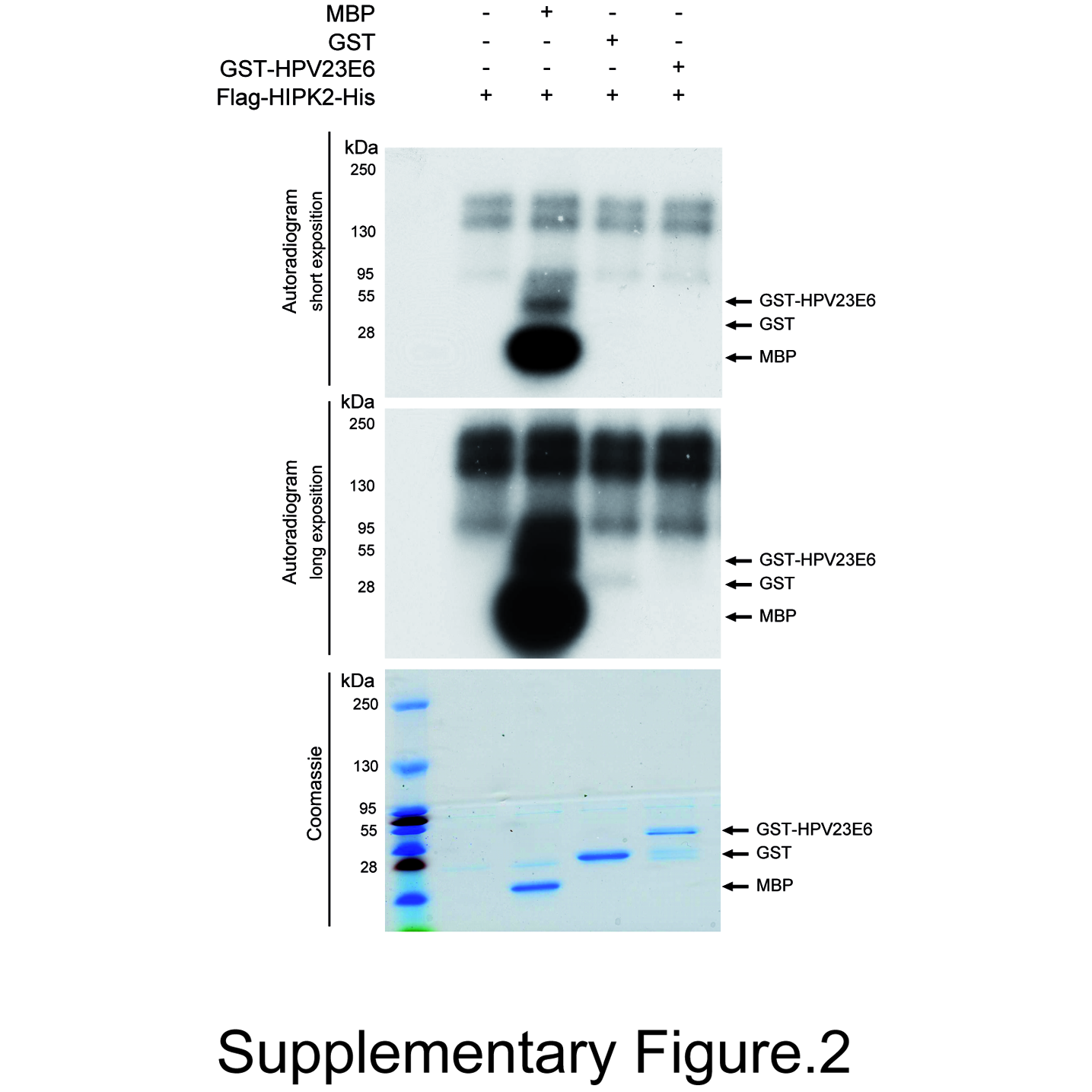

Supplement: Figure S2 — HIPK2 does not phosphorylate HPV23 E6. Autoradiogram showing an in vitro HIPK2 kinase assay using GST-tagged HPV23 E6 protein. The target protein MBP of HIPK2 was used as a positive control and GST as a negative control. Phosphorylated MBP is shown in short and long exposition whereas GST and HPV23 E6 was not phosphorylated by HIPK2 under these conditions. The protein loading was monitored by Coomassie blue staining (lower panel). (TIF) [file pone.0027655.s002.tif]
